# miR-335 negatively regulates osteosarcoma stem cell-like properties by targeting POU5F1

**DOI:** 10.1186/s12935-017-0398-6

**Published:** 2017-02-17

**Authors:** Xiaodong Guo, Ling Yu, Zhengpei Zhang, Guo Dai, Tian Gao, Weichun Guo

**Affiliations:** 1 Department of Orthopedics, Renmin Hospital of Wuhan University, Wuhan, Hubei China; 2 Key Laboratory of Carcinogenesis and Translational Research, Ministry of Education, Department of Orthopedic Oncology, Peking University Cancer Hospital & Institute, Beijing, China

**Keywords:** Osteosarcoma, Cancer stem-like cells, microRNA, POU5F1, Targeted therapy

## Abstract

**Background:**

Evidence is accumulating to link cancer stem cells to the pathogenesis and progression of osteosarcoma. The aim of this study is to investigate the role of miR-335 in osteosarcoma stem cells.

**Methods:**

Tumor spheroid culture and flow cytometry were applied to screen out osteosarcoma stem cells. Real-time quantitative PCR was used to detect the expression level of miR-335 in MG63, U2OS and 143B osteosarcoma stem cells. The relationship of miR-335 expression with osteosarcoma stem cells was then analyzed. Transwell assay and transplantation assay were performed to elucidate biological effects of miR-335 on cell invasion and vivo tumor formation. Western Blot and luciferase assays were executed to investigate the regulation of POU5F1 by miR-335.

**Results:**

The expression of miR-335 in osteosarcoma stem cells was lower than their differentiated counterparts. Cells expressing miR-335 possessed decreased stem cell-like properties. Gain or loss of function assays were applied to find that miR-335 antagonist promoted stem cell-like properties as well as invasion. Luciferase report and transfection assay showed that POU5F1 was downregulated by miR-335. Pre-miR-335 resulted in tumor enhanced sensitivity to traditional chemotherapy, whereas anti-miR-335 promoted chemoresistance. Finally, the inhibitory effect of miR-335 on in vivo tumor formation showed that combination of pre-miR-335 with cisplatin further reduced the tumor size, and miR-335 brought down the sphere formation capacity induced by cisplatin.

**Conclusions:**

The current study demonstrates that miR-335 negatively regulates osteosarcoma stem cell-like properties by targeting POU5F1, and miR-335 could target CSCs to synergize with traditional chemotherapeutic agents to overcome osteosarcoma.

## Background

Osteosarcoma cells are highly aggressive and are widely recognized as the most common primary malignant bone tumor in adolescents [1]. Traditional chemotherapy is associated with systematic toxicity and is less selective, and approximately 10–25% of patients respond poorly to the current chemotherapy [2]. Therefore, effective targeted therapy of osteosarcoma is an urgent problem.

Cancer stem cells (CSCs) are defined as cells with a self-renewal ability that can generate heterogeneous cancer cells, which play a key role in recurrence, metastasis, and drug resistance [3]. Several studies have also confirmed the existence of osteosarcoma stem cells. Gibbs first reported that stem-like cells exist in osteosarcoma, which are capable of forming self-renewable tumor spheres. Honoki reported that ALDH1 in MG63 sarcospheres was significantly higher and that these cells were involved in multidrug resistance [4]. Adhikari et al. have identified mouse and human osteosarcoma stem cells using mesenchymal stem cell markers CD-117 and Stro-1. These markers were preferentially expressed in spheres and doxorubicin-resistant cells [5]. The concept of CSCs provides a theoretical basis for specific targeted therapy. Therefore, studies of the regulation of osteosarcoma stem cells are especially important.

miRNAs are short non-coding RNAs that regulate different aspects of post-transcriptional gene expression, miRNAs usually bind to the 3′-untranslated region (3′-UTR) of targeted mRNAs, to cause translational degradation or target repression and gene silencing [6]. Recently, increasing evidence has suggested that miR-335 plays a role in the regulation of cancer progression. For instance, miR-335 was reported to inhibit small cell lung cancer bone metastases via the IGF-IR and RANKL pathway [7]. It has also been reported that miR-335 targets Bcl-w as an invasion suppressor gene in ovarian cancer [8]. Heyn [9] reported that miR-335 is crucial for the BRCA1 regulatory cascade in breast cancer development. In osteosarcoma, it has been suggested that miR-335 suppresses tumors by regulating the ROCK1 gene [10]. However, the relationship between miR-335 and osteosarcoma stem cells remains unclear.

In the present study, we showed that the levels of miR-335 were downregulated in osteosarcoma stem cells. Moreover, cells expressing miR-335 possessed decreased stem cell-like properties. These effects were partially caused by regulation of miR-335 targeting POU5F1. Furthermore, we demonstrated that miR-335 increased chemosensitivity and inhibited in vivo tumor formation by targeting osteosarcoma stem cells.

## Methods

### Cell lines and cell culture

MG63, U2OS and 143B cells were purchased from China Center for Type Culture Collection (CCTCC). HEK293 cells were obtained from Shanghai Institute for Biological Sciences of the Chinese Academy of Sciences. All the cells were cultured in RPMI-1640 containing 10% FBS and 1% penicillin/streptomycin. Cells were propagated at 37 °C with 5% CO_2_ and 100% humidity. Cell viability was determined using trypan blue staining.

### Tumor spheroid culture

Cells were plated in six-well ultralow attachment plates (Corning) at a density of 5000 cells/well in RPMI-1640 supplemented with B27 Supplement (Invitrogen), 10 ng/mL human EGF (Sigma-Aldrich), and 10 ng/mL human bFGF (Sigma-Aldrich). Cells were incubated at 37 °C in a humidified atmosphere of 95% air and 5% CO_2_. Fresh aliquots of EGF and bFGF were added every other day. After culture for 2 weeks, colonies larger than 50 μm in size were regarded as sarcospheres and quantitated by inverted phase contrast microscopy.

### Flow cytometry

For analysis of cell surface markers, osteosarcoma cells were harvested and resuspended in PBS/0.5% normal rabbit serum (Sigma-Aldrich), and blocked on ice for 15 min. Cells were subsequently labelled with Alexa Fluor^®^ 647 anti-human Stro-1 antibody (BioLegend) and PE anti-human CD117 (c-kit) antibody (BioLegend) for 60 min and maintained on ice until analysis. The expression was assessed by flow cytometry and data were analyzed. The double positive (DP) and double negative (DN) cells were then sorted and collected using a Becton–Dickinson FACSort (San Jose).

In order to divide the cells into different subpopulations according to their miR-335 expression status, we incubated the cells with miR-335-5p Hu-Cy5 SmartFlare™ RNA Detection Probe (Millipore), overnight for 16 h. The fluorescence was then detected and cells were sorted into miR-335-high and miR-335-low subpopulation using a Becton–Dickinson FACSort (San Jose). The collected cells were then send for subsequent experiments.

### Luciferase assays

Basing upon the pMIR-REPORT vector the luciferase reporter was constructed (Ambion). Strands of the oligonucleotides of the POU5F1-3′-UTR containing the miR-335 binding site were subcloned into the pMIR-REPORT vector after being synthesized and annealed. Scrambled sequences were also subcloned into the same vector acting as negative control. The POU5F1-3′-UTR-miR-335 or control plasmid was transfected into the HEK293 cells using Lipofectamine 2000 (Invitrogen). Luminescence were assayed using the luminometer.

### Transfection assay

Anti-miR-335 and pre-miR-335 used in this study were synthesized and confirmed by Shanghai Sangon Biotech Co. Ltd. Scrambled RNA (100 nmol) was act as negative control. We transfected the pre-miR-335, anti-miR-335 and their negative controls into all three cell lines in 6-well plates (1 × 10^5^ cell per well) with 5 μL siPORT NeoFX transfection agent (Ambion) according to the manufacturer’s instructions. The cells were harvested for further experiments at the indicated time.

### Transwell assay

To test the cell invasion capability, we precoated 8 micron transwells with 10 μg/cm^2^ matrigel (BD Bioscience), then added 3 × 10^6^ cells into the upper chamber with serum-free medium. The medium containing 10% FBS was put into the lower chamber as chemo-attractant. Cells were allowed to invade for 24 h incubation. Remaining cells which did not invade through the pores were carefully wiped out. Matrigel membranes were fixed in 90% methanol and then stained with crystal violet solution. Five random fields were counted by an inverted microscope (Olympus) examination. Each experiment was repeated at least three times.

### qRT-PCR

Total RNAs including mRNAs and small RNA fraction were isolated from cells using TRIZOL reagent (Invitrogen), and reverse transcriptions were performed by Takara RNA PCR kit (Takara) according to the manufacturer’s instructions. Real-time PCR was performed using a SYBR Green detection system which the U6 small nuclear RNA and β-actin mRNA were used as internal controls. All the reactions were repeated in three times. Forward and reverse primers for miR-335 and U6 were 5′-TCAAGAGCAATAACGAAAAATGT-3′, 5′-GCTGTCAACGATACGCTACGT-3′; and 5′-CGCTTCGGCAGCACATATAC-3′, 5′-TTCACGAATTTGCGTGTCAT-3′; respectively. The primers for POU5F1 and β-actin mRNA were 5′-GAGTGAGAGGCAACCTGGAGAAT-3′, 5′-ACCGAGGAGTACAGTGCAGTGAA-3′; and 5′-GTCCACCGCAAATGCTTCTA-3′, 5′-TGCTGTCACCTTCACCG TTC-3′, respectively.

### Western Blot analysis

Total proteins from transfected cells were obtained according to the manufacturer’s instructions (Sigma). We resolved the proteins by 10% SDS-PAGE gel and transferred them onto the PVDF membrane (Millipore). After being blocked in 10% nonfat dried milk for 2 h, the blots were incubated with anti-POU5F1 (1:1000; TA324014, OriGene) or anti-Sox2 (1:700; TA321559, OriGene) at 4 °C overnight. After washing three times with TBST, the blots were incubated with secondary antibody at room temperature for 1 h. After washing three times with TBST, membranes were developed by using enhanced chemiluminescence. β-actin served as an endogenous control.

### Cell cytotoxicity assay

The cells in each well containing 100 μL medium were incubated with 10 μL cell counting kit-8 (CCK-8) at 37 °C for 2 h. The optical density (OD) of each well was then measured at 450 nm using a microplate reader.

### Animals and transplantation assay

To determine the in vivo tumorigenicity, BALB/C nude mice about 6 weeks old were purchased and maintained at the Center for Animal Experiment at Renmin Hospital of Wuhan University. Osteosarcoma cells (1 × 10^6^) transfected with the pre-miR-335 or vehicle construct were counted by trypan blue staining, and suspended in 10 μL of 50% Matrigel/PBS. The mice were randomly divided into four groups (Vehicle, Cisplatin, pre-miR-335 and cisplatin + pre-miR-335 group), with six mice in each group. For cisplatin treatment, the mice received subcutaneous injection of 5 mg/kg cisplatin per week for 6 weeks. Due to the transient effect of pre-miR-335 transfection, the mice received intratumoral injection with 4 μg of lipofectamine 2000-encapsulated pre-miR-335 every week for the last 4 weeks. Tumors were monitored as long as 6 weeks. Data were accumulated from at least three independent experiments.

### Statistical analysis

All data were expressed as the mean ± standard deviation (SD). Statistical analysis were made between two groups with the t test. One-way ANOVA followed by multiple comparisons among the means was used where more than three means were compared. P < 0.05 was considered as statistically significant.

## Results

### Expression of miR-335 is downregulated in osteosarcoma stem cells

**Fig. 1:**
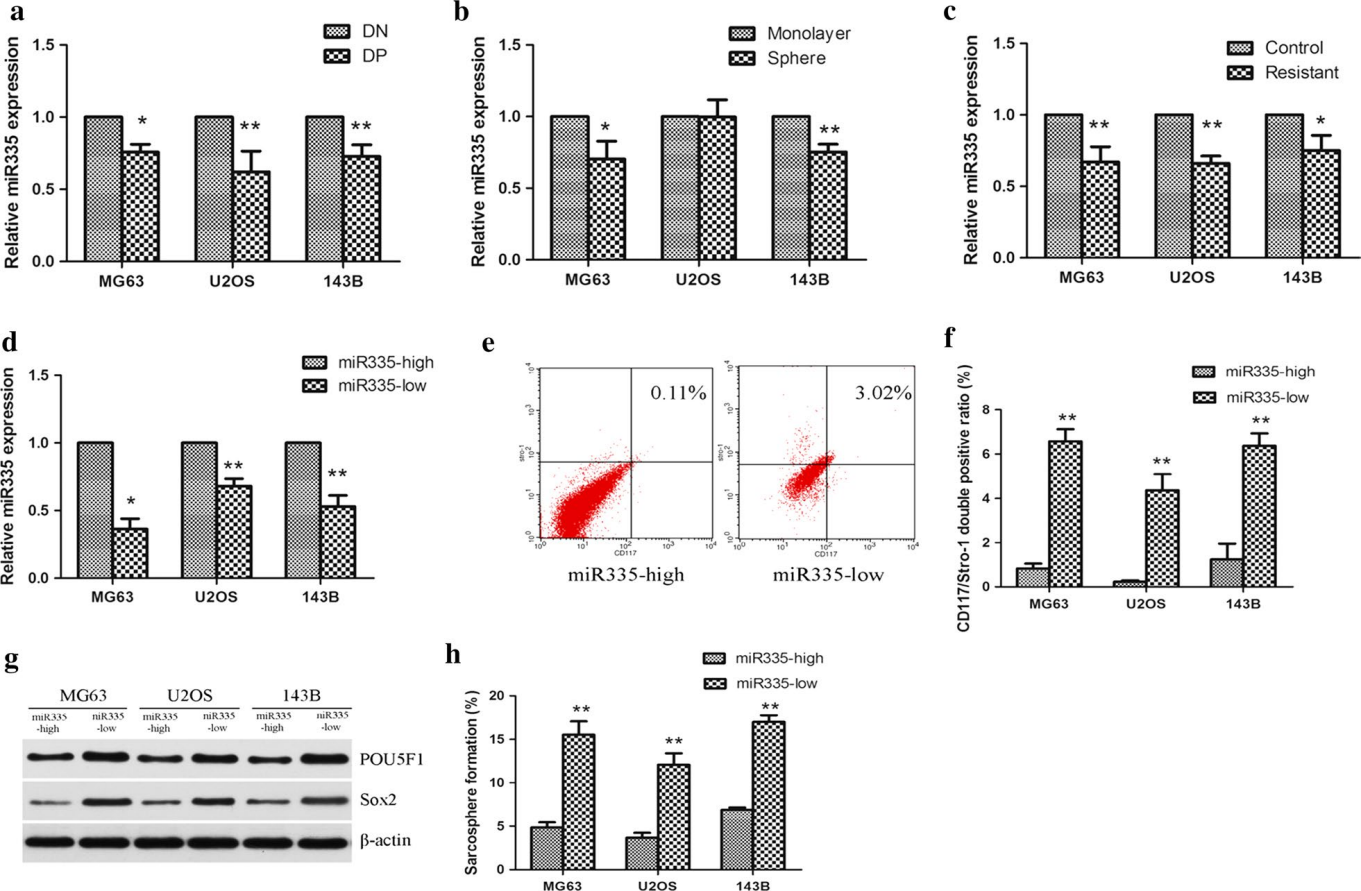
miR-335 was associated with osteosarcoma stem cells. **a** The relative expression of miR-335 were determined by qRT-PCR in CD117 and Stro-1 double positive (DP) cells and double negative (DN) cells. **b** miR-335 relative expression in sphere and monolayer culture conditions were examined. **c** The relative expression of miR-335 in the cisplatin resistant and control cells were tested. **d** The cells were divided into two groups according to their expression of miR-335. **e** A representative flow cytometric analyses of CD117+/Sro-1+ expression among miR-335-high and miR-335-low cells. **f** CD117+/Sro-1+ DP ratio in miR-335-low group was generally higher compared with miR-335-high in all three cell line. **g** Western-Blotting assay showed that POU5F1 and Sox2 expression were significantly decreased in miR-335-high compared with miR-335-low cells. **h** The ability to form sarcospheres was higher in miR-335-low compared with miR-335-high cells. All experiments were carried out at least triplicates and the data were presented as the mean ± SD. Student t test was performed to evaluate the difference. *P < 0.05; **P < 0.01

Because CD117 and Stro-1 double positive cells are thought to be enriched for osteosarcoma CSCs, we then compared miR-335 expression in double positive (DP) and double negative (DN) cell populations, and showed that the expression of miR-335 decreased significantly in DP cells in all three cell lines (Fig. 1a). CSCs are capable of forming spheres when cultured without serum and in an anchorage-independent manner, so we examined miR-335 expression in cells cultured under sphere-forming conditions. There was a significant decrease in the expression of miR-335 in sphere-cultured MG63 and 143B cells compared with their monolayer counterparts. This trend was also observed in U2OS cells although without statistical significance (Fig. 1b). Our previous study confirmed the selection of osteosarcoma CSCs by short-term cisplatin exposure [11]. In the present study, we further showed that the expression of miR-335 was significantly lower in resistant cells compared with control cells (Fig. 1c). Together, these results showed that miR-335 expression affected growth of osteosarcoma CSCs.

### Cells expressing miR-335 showed decreased stem cell-like properties

Based on the aforementioned results, we separated three osteosarcoma cell lines into miR-335-high and miR-335-low in order to compare their stem cell-like properties (Fig. 1d). CD117 and Stro-1 have been linked to cancer stem cells in osteosarcoma, so we determined whether miR-335-low cells were enriched with cells expressing these markers. The CD117 and Stro-1 double positive rate for MG63, U2OS and 143B miR-335-low cells were significantly higher than the miR-335-high cells (Fig. 1e, f). CSCs possess a transcriptional profile similar to that of embryonic stem cells [12]. The expression of the genes POU5F1 and Sox2, were significantly downregulated in miR-335-high cells compared with miR-335-low cells (Fig. 1g). Spheroid formation has been widely used to assess the in vitro self-renewal potential of stem cell-like cells, so we compared the ability of miR-335-low and miR-335-high cells to form sarcospheres. The miR-335-low cells formed more sarcospheres than miR-335-high cells in all three osteosarcoma cell lines (Fig. 1h). These results suggested that miR-335 negatively correlated with osteosarcoma stem cell-like properties.

### Downregulation of miR-335 enhanced osteosarcoma stem cell-like characteristics

**Fig. 2:**
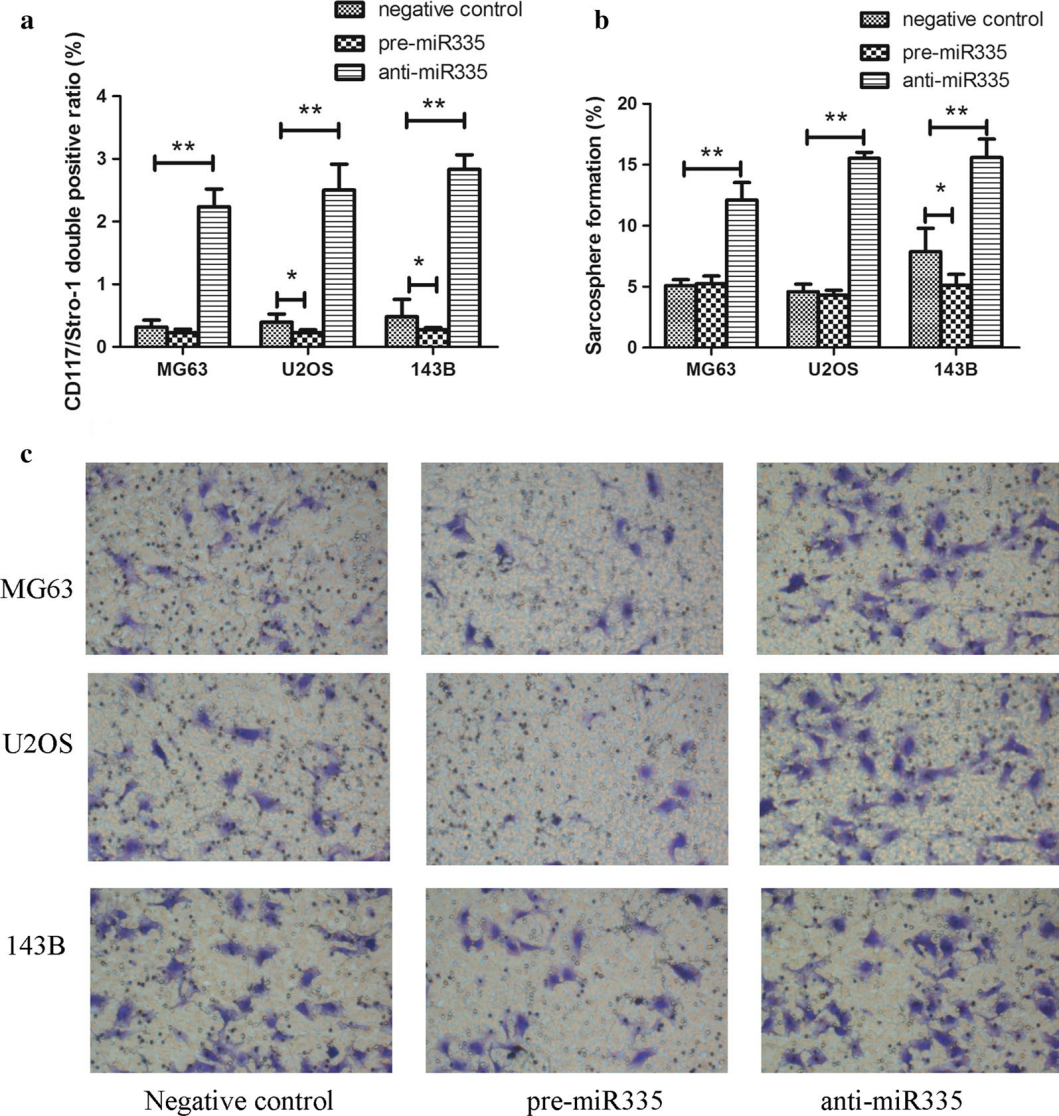
Downregulation of miR-335 enhanced osteosarcoma stem cell-like characteristics. **a** miR-335 antagonist increased CD117/Stro-1 double positive ratio in all three cell lines. **b** The ability of cells treated with anti-miR-335 to form sarcosphere was higher compared with pre-miR-335 and negative control cells. **c** Transwell invasion assay demonstrated that anti-miR-335 promoted cell invasion while pre-miR-335 inhibited it. All experiments were carried out at least triplicates and the data were presented as the mean ± SD. Student t test was performed to evaluate the difference. *P < 0.05; **P < 0.01

To confirm the effect of miR-335 on the stem cell-like properties of osteosarcoma cells, all three cell lines were transfected with pre-miR-335, anti-miR-335, or their negative controls respectively. The DP ratios of both CD117/Stro-1 and sarcosphere formation were significantly increased when exposed to a miR-335 antagonist, whereas pre-miR-335 inhibited the DP ratio and sarcosphere formation efficiency when compared with negative controls (Fig. 2a, b). CSCs are closely related to invasion and metastasis, so we determined the effect of miR-335 on cell invasion. The Transwell invasion assay showed that anti-miR-335 treatment of all three cell lines increased invasion of osteosarcoma cells when compared to negative controls, while pre-miR-335 inhibited cell invasion (Fig. 2c). Taken together, the results suggested that miR-335 was a suppressor of osteosarcoma stem cells.

### miR-335 negatively regulated POU5F1 gene expression

**Fig. 3:**
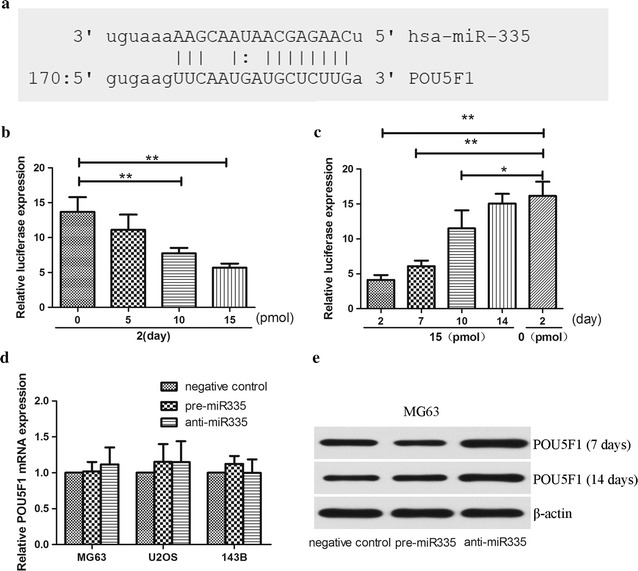
miR-335 negatively regulated POU5F1 gene expression. **a** Bioinformatics study indicated that miR-335 was partially complementary to the 3′-UTR of POU5F1 mRNA. **b** Different doses of pre-miR-335 were co-transfected with the specific pMIR-REPORT construct into HEK293 cells. Luciferase activities were measured and normalized to the phRL-TK activities. **c** The effect of pre-miR-335 gradually diminished in 2 weeks. **d** Osteosarcomas cells were transfected with pre-miR-335, anti-miR-335 or their control respectively. After induction for 7 and 14 days, the cells were harvested for measurement of POU5F1 mRNA using qRT-PCR and **e** the protein expression using Western blot. Untreated cells was set as control, and β-actin acts as an internal control. All experiments were carried out at least triplicates and the data were presented as the mean ± SD. Student t test was performed to evaluate the difference. *P < 0.05; **P < 0.01

Using Target Scan tools to search the target gene for miR-335, the POU5F1 3′-UTR was found to contain a putative target sequence for miR-335 (Fig. 3a). To further confirm if miR-335 binds directly to the 3′UTR of POU5F1, a pMIRREPORT Luciferase reporter was used and the POU5F1-3′-UTR-miR-335 reporter plasmid with the miR-335 mimic or its control were co-transfected into HEK293 cells. The relative luciferase activity was significantly decreased in a dose-dependent manner in HEK293 cells after treatment with pre-miR-335 (Fig. 3b). In addition, the effect of the transfected oligoribonucleotides diminished gradually. There was no obvious difference between the transfected and control group 2 weeks after transfection (Fig. 3c). Together, these results showed that miR-335 suppressed expression of transcripts containing a miR-335-binding site. To demonstrate whether miR-335 inhibited POU5F1 protein expression, all three cell lines with pre-miR-335, anti-miR-335 and their negative controls were transfected for 24 h. We tested the expression of POU5F1 in mRNA level and protein levels. Using qRT-PCR, there was no significant decrease or increase in the mRNA level of POU5F1 by qRT-PCR when compared with negative controls (Fig. 3d). In contrast, POU5F1 protein levels decreased after overexpression of miR-335 on day 7. It also significantly increased after knockdown of endogenous miR-335. These changes disappeared on day 14, which was probably due to the degradation of transfected oligoribonucleotides (Fig. 3e). Altogether, these results suggested that miR-335 potentially repressed only the protein expression of POU5F1.

### miR-335 decreased the in vivo tumor formation by targeting CSCs

**Fig. 4:**
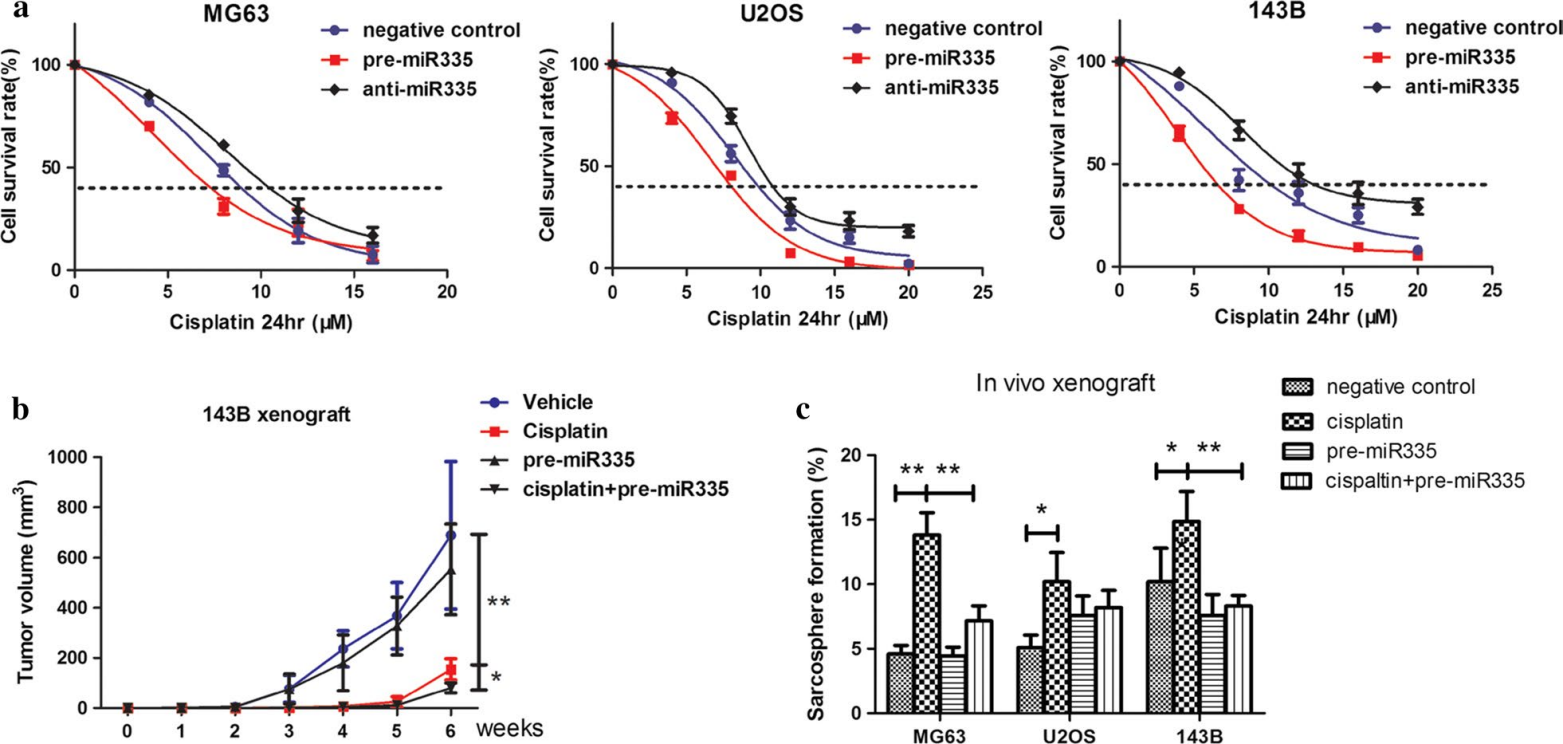
miR-335 decreased in vivo tumor formation by targeting CSCs. **a** Anti-miR-335 treatment promoted chemoresistance while pre-miR-335 enhanced sensitivity in all three cell lines. **b** Combination of cisplatin and pre-miR-335 orchestrated to reduce the tumor volume. **c** Pre-miR-335 treatment brought down the sarcosphere formation ability induced by cisplatin. All experiments were carried out at least triplicates and the data were presented as the mean ± SD. Student t test was performed to evaluate the difference. *P < 0.05; **P < 0.01

CSCs are thought to be resistant to traditional chemotherapy. To examine whether miR-335 affected the survival to current treatments, we used cisplatin. This compound, which is one of the most widely used reagents for osteosarcoma chemotherapy, was used to test the sensitivity of different osteosarcoma cells. We found that pre-miR-335 treatment enhanced the sensitivity in all three cell lines, whereas anti-miR-335 group promoted chemoresistance (Fig. 4a). We then determined the effect of pre-miR-335 on in vivo tumor formation. The mice were treated with pre-miR-335 and cisplatin, alone or in combination. On the sixth week, the tumors in the control mice were ~1 cm^3^, and pre-miR-335 alone did not alter the tumor growth. While cisplatin showed strong inhibitory effect on in vivo tumor formation, its combination with pre-miR-335 further reduced the tumor size (Fig. 4b). To investigate whether miR-335 targeted cancer stem cells in vivo, xenografts from each group were obtained and assayed for their sphere formation efficiency. Cisplatin alone increased the capacity to form spheres. Although miR-335 treatment alone did not affect the sphere formation capacity, it decreased the sphere formation induced by cisplatin (Fig. 4c). Together, these results suggested that miR-335 targeted CSCs in a synergistic manner with cisplatin, to inhibit growth of osteosarcoma.

## Discussion

Accumulating evidence has indicated that miRNAs act as key regulators of cell proliferation, differentiation and apoptosis [13]. MicroRNAs have been reported to play roles in the maintenance and regulation of normal and malignant stem cells [14]. Our results showed that miR-335 negatively regulated osteosarcoma stem cell-like properties. First, we showed that the expression of miR-335 in osteosarcoma stem cells was lower than their differentiated counterparts. Second, cells expressing miR-335 possessed decreased stem cell-like properties compared with miR-335 low cells. Third, the miR-335 antagonist promoted stem cell-like properties, whereas miR-335 expression showed the opposite effect. Fourth, we showed that pre-miR-335 treatment reduced tumor cell invasion and resistance, whereas anti-miR-335 promoted tumor cell invasion and resistance. Fifth, we showed that miR-335 decreased the stem cell-like properties induced by cisplatin as assessed by the sphere formation efficiency.

Osteosarcoma is thought to derive from mesenchymal stem cells (MSCs) [15]. miR-335 has been reported to induce cell proliferation, migration and differentiation in human MSCs [16]. Previous studies have also reported that miR-335 is downregulated in osteosarcomas [10, 17]. However, the status of miR-335 in osteosarcoma stem cells remains to be elucidated. miR-335 was shown to be downregulated in breast cancer stem cells and to inhibit CSC growth [18]. In the present study, three different methods were used to isolate osteosarcoma stem cells, and the expression of miR-335 was obviously down-regulated in osteosarcoma stem cells. Additionally, functional experiments showed that downregulation of miR-335 promoted osteosarcoma cells stem cell-like properties. These results were consistent with those of Schoeftner’s study, which showed that miR-335 controlled the Oct4-pRb axis and integrated stem cell self-renewal and cell cycle control [19].

Previous studies have reported that POU5F1 plays an extremely important role in maintaining the cancer stem cell fate of osteosarcoma [20]. Characterizing mechanism of POU5F1 regulation is therefore of vital interest. In the present study, we showed that POU5F1 gene expression was negatively regulated by miR-335. The Target Scan software [21] predicted that the POU5F1 3′-UTR was incompletely bound to miR-335, which means that it could repress POU5F1 expression by post-translational regulation. Consistent with this possibility, when we fused the miRNAs regulatory elements (MREs) from the POU5F1 3′-UTR to luciferase, the luciferase reporter analysis showed that exogenous pre-miR-335 and anti-miR-335 regulated the activity, and that suppression occurred in a dose-dependent manner. We further observed that POU5F1 was negatively regulated by miR-335 merely at the protein level through gain- or loss- of function studies. These results further confirmed that miR-335 repressed POU5F1 expression via imperfect complementation to the POU5F1 mRNA 3′-UTR.

CSCs possess intrinsic chemoresistance. The underlying mechanisms include higher levels of the ABC transporter and anti-apoptosis genes, efficient DNA repair, and detoxification by aldehyde dehydrogenase. In addition, CSCs express EMT markers suggesting that they are able to migrate [22, 23]. Consistent with these results, our studies showed that miR-335 significantly decreased drug resistance compared with the anti-miR-335 group and with the control group. Accordingly, the present study showed that miR-335 significantly inhibited the invasive potential of metastasizing cells.

CSC-targeted therapy is a promising procedure to treat tumor cells. We therefore investigated whether miR-335 could selectively inhibit CSCs. Cisplatin alone increased the capacity to form tumor spheres in a osteosarcoma xenograft model, which was consistent with our previous results that conventional chemotherapy enriched for CSCs [11]. However, miR-335 worked together with cisplatin in shrinking the tumor size, as well as decreasing the sphere formation capacity induced by cisplatin. These results suggested that miR335 could be an effective agent to eradicate CSCs.

Nonetheless, there remain some limitations in the current study. Firstly, we did not provide clinical relevance in our study due to the scarcity of osteosarcoma stem cell in human samples. Testing the inhibitory effect of pre-miR-335 on patient-derived xenograft could thus provide more clinical importance. Secondly, it is of great interest to investigate the role of miR-335 in other malignant bone tumor such as Ewing sarcoma and chondrosarcoma. Thirdly, there are plenty of genes targeted by miR-335, it is unknown whether genes except POU5F1 also participate in regulating osteosarcoma stem cells. Finally, we used oligoribonucleotides transfection to regulate miR-335 expression, however, the effect diminished gradually, pre- or anti-miR-335 inducible stable cell lines could provide stronger evidence and further support to our findings.

## Conclusions

Taken together, our results for the first time showed the relationship between miR-335 and POU5F1 in osteosarcoma stem cells. Aside from the above limitations, these findings will characterize the underlying mechanisms of osteosarcoma and may facilitate the development of novel therapeutic strategies for clinical application.

## Abbreviations

miR: microRNA
CSCs: cancer stem cells
POU5F1: POU domain, class 5, transcription factor
qRT-PCR: quantitative real-time polymerase chain reaction
EMT: epithelial-mesenchymal transition
